# Management of diabetes mellitus in patients with severe mental disorders using new generation hypoglycemic drugs- A review of the literature

**DOI:** 10.1192/j.eurpsy.2023.1226

**Published:** 2023-07-19

**Authors:** O. Vasiliu

**Affiliations:** Psychiatry Department, Dr. Carol Davila University Emergency Central Military Hospital, Bucharest, Romania

## Abstract

**Introduction:**

Severe mental disorders (i.e., schizophrenia spectrum disorders and bipolar disorders) have been associated with a high incidence of metabolic dysfunctions, diabetes mellitus (DM) included. There are multiple factors converging to this phenomenon, and not all of them are yet known (i.e., a specific genetic vulnerability, lifestyle factors, adverse events of antipsychotics and antidepressants, etc.). Glucagon-like peptide 1 receptor agonists (GLP1RAs), dipeptidyl-peptidase-4-inhibitors (DPP4Is), and sodium-glucose cotransporter 2 inhibitors (SGLT2Is) are new hypoglycemic drugs which are generally well tolerated and associate good glycemic control in clinical trials (usually in combination with classical antidiabetics).

**Objectives:**

To explore the available evidence supporting the use of new-generation hypoglycemic drugs (NGHD) in patients with severe mental disorders with comorbid DM.

**Methods:**

A literature review was performed through the main electronic databases (PubMed, CINAHL, Clarivate/Web of Science, and EMBASE) using the search paradigm “schizophrenia spectrum disorders” OR “bipolar disorders” OR “major depression” AND “diabetes mellitus” AND “new-generation hypoglycemic agents” OR “glucagon-like peptide 1 receptor agonists” OR „dipeptidyl-peptidase-4-inhibitors” OR „sodium-glucose cotransporter 2 inhibitors”. All papers published between January 2000 and September 2022 were included.

**Results:**

Based on the reviewed papers, GLP1RAs may be useful (n=20 sources identified) in order to obtain glycemic control in patients with severe mental disorders receiving antipsychotics; SGLT2Is added to metformin could be beneficial to the same population, but data that support their use is extremely limited (n=4 sources); extremely limited data (n=2) about DPP-4Is do not allow to formulate any recommendation about this class in patients with severe mental disorders and associated DM. A number of ongoing trials have also been identified during this search (n=5), especially focused on GLP1RAs, which are expected to bring more information regarding the efficacy and safety of these drugs in this specific population. Most of the collected data in this review were of low and moderate quality.

**Conclusions:**

Based on the currently available evidence, GLP1RAs and SGLT2As may be useful in the management of DM in patients with severe mental disorders, but more data about their long-term efficacy and safety is required before making any categorical recommendation.

**Disclosure of Interest:**

None Declared

